# Evaluating Physical and Tactical Performance and Their Connection during Female Soccer Matches Using Global Positioning Systems

**DOI:** 10.3390/s23010069

**Published:** 2022-12-21

**Authors:** Ibai Errekagorri, Ibon Echeazarra, Aratz Olaizola, Julen Castellano

**Affiliations:** 1 Department of Physical Education and Sport, Faculty of Education and Sport, University of the Basque Country (UPV/EHU), Lasarte 71, 01007 Vitoria-Gasteiz, Álava, Spain; 2 Society, Sports and Physical Exercise Research Group (GIKAFIT), Department of Physical Education and Sport, Faculty of Education and Sport, University of the Basque Country (UPV/EHU), Lasarte 71, 01007 Vitoria-Gasteiz, Álava, Spain; 3 Department of Didactics of Corporal Expression, Faculty of Education and Sport, University of the Basque Country (UPV/EHU), Juan Ibáñez de Santo Domingo 1, 01006 Vitoria-Gasteiz, Álava, Spain

**Keywords:** team sport, women’s football, match analysis, time-motion analysis, electronic performance and tracking systems

## Abstract

The objective of the present study was to evaluate the tactical and physical performance during official matches of a women’s soccer league and to correlate both dimensions in periods of 15 min. To do this, eight official matches of a semi-professional soccer team belonging to the Women’s Second Division of Spain (Reto Iberdrola) were analysed during the 2020–2021 season. The variables recorded were classified into two dimensions: tactical variables (i.e., Width, Length, Height and Surface Area) and physical variables (i.e., Total Distance Covered (TD), Total Distance Covered in High-Speed Running (HSR) and Total Distance Covered in Sprint). The main results were: (1) there were no differences between the periods in any of the tactical dimension variables; (2) in the physical dimension, a significant decrease in TD and HSR was described at the end of the match (period 60–75); and (3) some positive correlations were found among some variables of the tactical and physical dimension at the beginning and at the end of the match (periods 0–15, 60–75 and 75–90). The findings of the study suggest that connecting the tactical and physical dimension in the interpretation of team performance would allow for a better understanding of player and team performance and during competition.

## 1. Introduction

Due to technological advances available in positional monitoring of soccer players (i.e., electronic performance and tracking systems), greater opportunities have been generated to approach, with scientific rigor, the analysis of the activity of athletes, during both training and competition [1]. Although it is true that these technologies have been used predominantly in applied research environments, such as the domain of space-time analysis, focusing especially on describing the physical or conditional performance of players [2], each time there is more research that tries to identify, from players’ positioning, the structure of the team and its evolution as the game progresses, being able to describe and explain the adaptive dynamics of a group of players (or the total) during a match [3,4,5,6].

The physical dimension has been studied in greater depth than other dimensions during soccer competitions [1,7]. There are several studies that quantified the physical characteristics of the players for each 15 min period of time, where it was observed, on the one hand, that the first 15 min of the match was consistently the most demanding period, and on the other, a performance decrease in variables, such as total distance covered, high-speed running or sprinting as the game progressed, especially in the last 15 min period [8,9,10,11,12,13]. This could be justified by signs of fatigue or the inability to consistently perform at the same level throughout the course of the match, even at the highest standards of the professional arena [14]. However, some authors [15] suggest attributing the lower physical response to a decrease in the performance of the players caused by fatigue is perhaps an overly simplistic explanation, and other issues to achieve a better understanding of the physiological response during competition should be considered, which are, so far, limited. These authors note that female soccer players will rarely stop participating in a match prematurely due to exhaustion, and that this is likely to be regulated by the player and influenced by a number of contextual factors, in addition to a possible self-regulation of effort as a match progresses [16].

In soccer, performance is essentially tactical, determined by the proper coordination of the behaviour of the players on the field of play, and the implementation of technical skills to complete key actions of the game complements this movement, which, to a large extent, is what determines the physical response of the performance of athletes [7]. From a practical point of view, a soccer match must be understood on the basis of reciprocal relationships between the state of movement of the attack vs. defence phases of both teams in interaction [17]. In this sense, performance must be understood in terms of space-time interaction dynamics and not only in terms of individual time-motion demands of the players [18]. Therefore, the factors that determine performance should not be considered separately [14,19], since soccer is a complex activity that depends on multiple factors to optimize the performance of players and teams [20]. In this sense, there are works that have described several of these dimensions simultaneously (physical and tactical) in different male youth-soccer categories [21], where complex technical and physical indicators [22] or where physical variables with centrality metrics have been correlated [23]. However, there is no study in the scientific literature that connects physical variables with tactical variables.

Therefore, there is a need to jointly consider tactical and physical response for a holistic understanding of game performance in the field of women’s soccer [24]. Taking this into consideration, the objectives of this study were: (1) to evaluate tactical and physical performance during official women’s soccer league matches and (2) to correlate the tactical and physical dimension, considering periods of 15 min. There were two starting hypotheses: on the one hand, there would be differences in physical and tactical performance throughout the periods of the game and, on the other, there would be a negative correlation between both dimensions. Therefore, actions with a greater physical component (more accumulated distance) would imply less intervention on the ball and, therefore, lower values of the collective behaviour variables.

## 2. Materials and Methods

### 2.1. Study Design

This is an observational descriptive study, in which eight official matches of the Second Women’s Division of Spain (Reto Iberdrola) were analysed during the 2020–2021 season. The analysed matches were played during the competition period in the first phase (also named regular phase). In the eight matches analysed in the study, the team assessed won five and lost three; five matches were played at home and three away. Finally, four matches were played against opponents that qualified for promotion to the first division and the other four against opponents that played to stay up.

### 2.2. Participants

A total of 22 semi-professional soccer players (age: 24.6 ± 4.0 years; height: 163.9 ± 5.0 cm; body mass: 58.5 ± 4.2 kg) belonging to the same female soccer team participated in this study. The team regularly trained five 90 min sessions a week, plus a competitive match each weekend. To carry out the study, goalkeeper data were excluded.

The analysed team was a dominant team throughout the season, winning 18 games out of 24 played, thus, finishing first in the final table and being promoted to the First Women’s Division of Spain (Liga Iberdrola). This team was characterized as having a possession style with a high pass rate and occasionally (e.g., if the team is winning or playing away) with a direct style of play and high-pressure defending.

### 2.3. Variables

Similar to other studies [1,4,7,25], the following variables were recorded for each match and grouped into two dimensions. On the one hand, the tactical variables were: Width, Length, Height and Surface Area (SA). On the other hand, the physical variables were: Total Distance Covered (TD), Total Distance Covered in High-Speed Running (HSR) and Total Distance Covered in Sprint (Sprint). Table 1 shows the definitions of all variables used in this study for each dimension.

### 2.4. Procedures

In order to obtain position data to be able to calculate the variables, players were monitored with WIMU PRO devices (RealTrack Systems, Almería, Spain) using Global Positioning System (GPS) technology. The GPS devices used in this study can operate at 10 Hz and they are compatible with the Galileo and American satellite constellation, which seems to provide more precision [26]. For the analysis, the data were collected in outdoor soccer fields, without any possibility that infrastructures leave the data collection. During the games, a mean of 12 satellites was connected to each device. The value of DDOP was 0.95. This equipment and its measurements are valid and reliable using GPS for time-motion analysis in soccer (distance covered variable: accuracy = 0.69–6.05%, test-retest reliability = 1.47, inter-unit reliability = 0.25; mean velocity variable: accuracy = 0.18, intra-class correlation = 0.951, inter-unit reliability = 0.03) [27] and were awarded with the FIFA Quality Performance certificate. Additionally, the agreement of the data on the collective tactical variables during an official soccer match between global and local positioning systems (that has a very acceptable precision to estimate the position of the players on the pitch [28]) was tested with an intra-class correlation coefficient greater than 0.84 [29].

Relative values were used to analyse the speed data [30,31,32]. In order to obtain these relative values, data were collected from all the sessions (trainings and matches) of the analysed team over 17 weeks and, from there, the maximum value of each player was defined to establish the relative speed threshold for both HSR and Sprint (Table 1). Each WIMU PRO device was placed on a vertical position between the players’ shoulder blades, in a pocket of a specific chest vest (dimensions of the devices = 81 × 45 × 16 mm). The devices were activated 15 min before the warm-up of the match to avoid the so-called “technological lockout” [33,34]. During the entire registration period, each player wore the same WIMU PRO device.

The records were downloaded using the software SPRO 983 (RealTrack Systems, Almería, Spain) after the end of each match. Taking into account previous studies [12,35], the matches were divided into periods of 15 min. This division was utilised to have more and shorter periods of time that favour the comparison and, thus, respond to the purpose of the study. To facilitate the comparison, data collected in injury time were not included for analysis, as the duration of the respective match halves was never identical. In order to calculate the tactical variables from the players’ positions on the pitch, data were transformed into raw position data (latitude and longitude) using the software’s GIS (Geographic Information System) mapping application, which allows for all kinds of geometric shapes, such as polygons or circles, with millimetre precision. Once the data were filtered through the software, they were imported into a Microsoft Excel spreadsheet (Microsoft Corporation, Redmond, WA, USA) to configure a matrix.

### 2.5. Statistical Analysis

Descriptive statistics data from variables were presented using mean and standard deviation. Tests for normality (Shapiro–Wilk) and equality of variances (Levene) were applied. The null hypothesis was accepted because the distribution of the data met the normality criterion. Furthermore, the variances were homogeneous. Therefore, ANOVA repeated measures were performed to test for differences in the dependent variables (Width, Length, Height, SA, TD, HSR and Sprint) between the 15 min periods (0–15, 15–30, 30–45, 45–60, 60–75 and 75–90). Significant results were analysed using post hoc Tukey’s test. The effect size was calculated using generalized eta squared (η^2^G). A generalized eta squared effect size of η^2^G = 0.01 was considered a small effect size; an effect size of η^2^G = 0.06 was considered a medium effect size; and an effect size of η^2^G = 0.14 was considered a large effect size [36]. In addition, a Pearson’s r correlation analysis was implemented between tactical and physical dimension for each 15 min period. As proposed by Hopkins [37], the following qualitative correlation descriptors were used: trivial (0–0.09), small (0.1–0.29), moderate (0.3–0.49), large (0.5–0.69), very large (0.7–0.89), nearly perfect (0.9–0.99) and perfect (1). The level of significance was set at *p* < 0.05. The statistical analysis was conducted using the software JASP 0.16.4 (University of Amsterdam, Amsterdam, Kingdom of the Netherlands) for Windows.

## 3. Results

**Figure 1:**
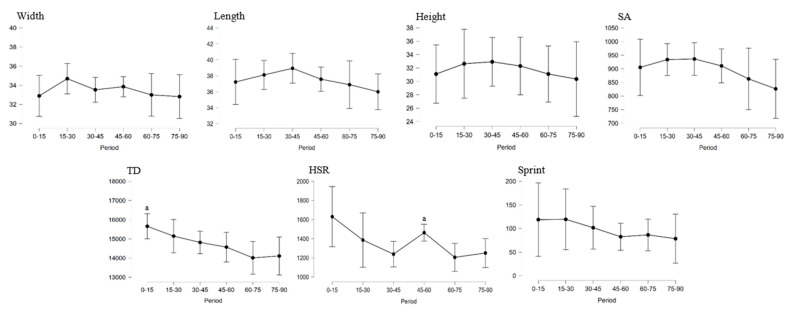
Mean and standard deviation of the variables for each period. Width is the mean team width, Length is the mean team length, Height is the mean team defence depth, SA is the mean team surface area, TD is the total distance covered and accumulated, HSR is the total distance covered and accumulated above the high-speed running relative speed threshold (60% of the player’s maximum speed) and Sprint is the total distance covered and accumulated above sprint relative speed threshold (85% of the player’s maximum speed). a > 60–75 for a significance level of *p* < 0.05.

Figure 1 shows the means and standard deviations of the tactical and physical variables for periods of 15 min. The periods had a significant large effect on TD (F = 3.45; *p* = 0.012; η^2^G = 0.276) and HSR (F = 3.63; *p* = 0.010; η^2^G = 0.229). In the period 0–15 of the match, the TD variable showed higher values than in the period 60–75 (mean differences: 1643.6 m; *p* = 0.045). Higher values of HSR were also observed in the period 45–60 of the match compared to the period 60–75 (mean differences: 257.8 m; *p* = 0.032).

Table 2 shows the values of Pearson’s r correlation between the variables of the tactical and physical dimension for each period. First, a very large positive correlation was found between Width and TD (r = 0.84; *p* = 0.010; 95% CI (0.318–0.969)), between Length and HSR (r = 0.73; *p* = 0.040; 95% CI (0.049–0.947)) and between SA and TD (r = 0.78; *p* = 0.022; 95% CI (0.175–0.959)) in the period 0–15 of the match. Second, a very large positive correlation was found between Width and HSR (r = 0.79; *p* = 0.019; 95% CI (0.200–0.961)) and between SA and HSR (r = 0.75; *p* = 0.034; 95% CI (0.085–0.951)) in the period 60–75. Third and last, a very large positive correlation was found between Height and Sprint (r = 0.85; *p* = 0.008; 95% CI (0.353–0.972)) in the period 75–90 of the match.

## 4. Discussion

The objective of the study was to describe the tactical and physical performance during official women’s soccer matches and, at the same time, to check whether they correlated, taking into account the different periods of the match. To the authors’ knowledge, this is the first study to analyse the connection between the tactical and physical dimension during competitions in women’s soccer. The results suggest some peculiarities in the evolution and relationship between both dimensions during women’s soccer matches: (1) there were no differences between periods in any of the variables of the tactical dimension; (2) in the physical dimension, a significant decrease in TD and HSR was described at the end of the match (period 60–75); and (3) some positive correlations were found between some variables for the tactical and physical dimension at the beginning and at the end of the match (periods 0–15, 60–75 and 75–90). Considering the results of the present study, the first hypothesis was partially confirmed: hardly any significant decrease in physical performance as the match progressed was found, whereas no significant differences were found in the tactical dimension. However, the second hypothesis was not confirmed, as there were some positive correlations between some variables of both dimensions in different periods.

Regarding the physical dimension, the results of this study showed a significant decrease in the TD variable as the match progressed, obtaining the highest values in the first period of the match (0–15 (15,658.1 m)). This period (0–15) showed significantly higher values than the period 60–75 (14,014.5 m). Although there were hardly any differences, this result is consistent with those of previously published works on the physical demands in female soccer competitions, where the highest values of the TD variable are in the first periods and they decrease as the game progresses [8,10,11,13]. On the other hand, the HSR variable showed significantly higher values in the period 45–60 (1463.4 m) compared to the period 60–75 (1205.5 m) of the match. This result coincides, in part, with those obtained in previous investigations [8,9,10,11,12], where a decrease in the HSR variable was described as the match progressed, mainly in the last two 15 min periods of the match. Regarding the Sprint variable, there was no significant decrease as the match progressed, an aspect that does not coincide with those obtained in previous investigations [8,9,10,11,12], where a decrease in physical performance was described in this variable, mainly in the last two 15 min periods of the match. The interpretation of these apparently contradictory results could be justified considering that the players were selective in their physical response, according to the context of the match, modified by situation variables, such as the momentary result [38]. In this way, resources are guaranteed to better solve the key moments and, probably, the most demanding ones in a match (e.g., maintain distance covered at high or very high speed), while in others, the pauses are lengthened and the physical response reduced (e.g., decrease in total distance covered).

Regarding the tactical dimension, it should be noted that, despite an upward trend in the first part and a downward trend in the second, there were no significant differences in the average values of the match periods in any of the tactical variables. The team showed an increase in the values of width, length and height of the defence from the start of the match until the break. In the second half, the team tended to play more compactly and with the height of the defence closer to their goal as the match progressed. One understanding is to do with the superiority shown by the team during the season (i.e., they won the league), which, on many occasions, having taken the lead in the last part of the matches, caused the rival to take the initiative in the match. The rival, especially, but also the situation variables, are part of the activity of playing a soccer match [14,38]. For this reason, and as this study reflects, the teams condition their behavioural and physical performance, trying to respond as efficiently as possible in each context that occurs during a match [38].

A multilevel and multidimensional approach, that is, from the individual to the team passing through intermediate groupings (e.g., dyads, system lines…), integrating tactical, physical, behavioural, emotional dimensions, etc., is essential in interpreting the performance of the teams in competition [39]. In this attempt to connect dimensions, the results of this study show that some variables of the tactical and physical dimension were positively correlated at some moments of the match, mainly in the first and last two periods of the match. Thus, for example, in the first period of the first part (0–15), the variables Width and SA were correlated with TD and Length with HSR. It seems that the intention of the team at the beginning of the match was to combine possession soccer with direct play. On the one hand, their intention was to maintain possession of the ball with a high pace of play while making wide and large collective use of the space on the pitch. On the other hand, it seems that they tried to complement control of possession with a more direct style by making high-speed movements playing deep. In the fifth period (60–75), Width and SA correlated with HSR. In the last period of the match (75–90), Height was correlated with the variable Sprint. In the penultimate period of the match, it seems that the team switched to high-speed action while making large and wide collective use of space. In turn, in the last period of the match, it seems that they began to perform Sprint actions moving the defensive line forwards. This may represent a counterattack game due to playing against a top team who, perhaps because of going behind, had to come back, defending up front, leaving free spaces behind. Further, these results indicate that the physical response of the team could be intimately connected with the tactical decisions of the team.

This study is not without limitations. In the first place, it represents an aprioristic study where the results obtained may depend on the specific characteristics and the particular tactical behaviour of the team analysed. Therefore, the sample used, which was not very large, tells us of the need to be cautious when extrapolating the results obtained to other teams or to female soccer players in general. It would be interesting for future research, based on similar methodology analysis, to compare the performance of different female soccer teams in competition, in order to identify factors that determine or contribute to success. The second limitation has to do with the impossibility of having the recordings of the matches (not allowed in professional soccer) to complement it with a notational analysis. From the video, it would have been possible to distinguish, for example, when the ball was in play or not, and even detailing possession for each team in each period. It is known that the physical [38] and tactical [40] responses of the teams differ when the team has or does not have possession of the ball and, probably, it would have allowed one to describe more precisely how both dimensions are connected [38]. This subject, distinguishing the attack and defence phase, is suggested for future research. The third limitation of the study was the non-inclusion of contextual variables due to a small sample being used. The choice of these kinds of variables, such as the place where the match takes place (home or away), the match outcome, the match status or the quality of the opponent, could have provided contextual information and another possible interpretation of the results of this study. Therefore, future research should consider different situational variables.

## 5. Conclusions

The present work is a novel proposal, since it not only evaluates the tactical and physical performance of the team as the match progresses, but also tries to describe if there is a correlation between both dimensions. The main conclusions of this study are: (1) physical and tactical performance evolved in a particular way throughout the match and (2) the way of approaching the analysis to assess the connection between both dimensions was unable to find strong correlations throughout the match. It should be noted that more research is needed to evaluate the tactical and physical demands of women’s soccer teams simultaneously, in order to improve the understanding of the game based on the connection between dimensions.

## Figures and Tables

**Table 1:** Definitions of the variables for each dimension.

Dimension	Variable	Definition
Tactical dimension	Width	Mean team width per match, understood as the distance in meters (m) between the two players furthest-apart across the width of the pitch [4,25].
	Length	Mean team length per match, understood as the distance in meters (m) between the two players furthest-apart along the length of the pitch [4,25].
	Height	Mean team defence depth per match, understood as the distance in meters (m) between the furthest back player and the goal being defended [4,25].
	SA	Mean team surface area per match, understood as total square meters (m^2^) of a polygon described by players as its vertex point and calculated using the convex hull calculation [4,25].
Physical dimension	TD	Total distance covered and accumulated in meters (m) by all the pitch players in the team that participated in the match [1,7].
	HSR	Total distance covered and accumulated in meters (m) above the high-speed running relative speed threshold (60% of the player’s maximum speed) by all the pitch players in the team that participated in the match [1,7].
	Sprint	Total distance covered and accumulated in meters (m) above the sprint relative speed threshold (85% of the player’s maximum speed) by all the pitch players in the team that participated in the match [1,7].

**Table 2:** Pearson’s r correlation values between tactical and physical dimension for each period.

	0–15				15–30		
	TD	HSR	Sprint		TD	HSR	Sprint
**Width**	0.84 *	0.63	0.37	**Width**	0.62	−0.07	−0.47
**Length**	0.69	0.73 *	0.64	**Length**	0.12	0.37	0.25
**Height**	0.64	0.65	0.21	**Height**	0.64	−0.19	−0.63
**SA**	0.78 *	0.64	0.54	**SA**	0.45	0.09	−0.28
	**30–45**				**45–60**		
	**TD**	**HSR**	**Sprint**		**TD**	**HSR**	**Sprint**
**Width**	0.38	0.05	0.52	**Width**	0.69	0.36	0.06
**Length**	0.08	0.59	0.04	**Length**	0.48	0.44	0.20
**Height**	0.01	0.11	−0.30	**Height**	−0.31	−0.09	0.53
**SA**	0.46	0.43	0.48	**SA**	0.66	0.26	−0.02
	**60–75**				**75–90**		
	**TD**	**HSR**	**Sprint**		**TD**	**HSR**	**Sprint**
**Width**	0.22	0.79 *	0.46	**Width**	0.04	0.36	0.67
**Length**	0.25	0.66	0.38	**Length**	−0.67	0.13	0.39
**Height**	0.36	0.64	0.35	**Height**	−0.58	0.54	0.85 *
**SA**	0.27	0.75 *	0.38	**SA**	−0.23	0.03	0.45

Note: Width is the mean team width, Length is the mean team length, Height is the mean team defence depth, SA is the mean team surface area, TD is the total distance covered and accumulated, HSR is the total distance covered and accumulated above the high-speed running relative speed threshold (60% of the player’s maximum speed) and Sprint is the total distance covered and accumulated above sprint relative speed threshold (85% of the player’s maximum speed). * is *p* < 0.05.
